# Results of an early intervention programme for patients with bacteraemia: analysis of prognostic factors and mortality

**DOI:** 10.1186/s12879-017-2458-x

**Published:** 2017-05-22

**Authors:** A. del Arco, J. Olalla, J. de la Torre, A. Blázquez, N. Montiel-Quezel, J. L. Prada, F. Rivas, J. García-Alegría, F. Fernández-Sánchez

**Affiliations:** 1 Infectious Diseses Group. Internal Medicine Unit. Costa del Sol Hospital, 29603 Marbella, Málaga Spain; 2 Microbiology Unit, Hospital Costa del Sol, Marbella, Spain; 3 Research Support Unit, Hospital Costa del Sol, Marbella, Spain; 4 Health Services and Chronic Disease Research Network (REDISSEC), Marbella, Spain

**Keywords:** Bacteraemia, Intervention programme, Prognosis, Mortality

## Abstract

**Background:**

Bacteraemia is a common cause of morbidity and mortality in patients admitted to hospital. The aim of this study is to analyse the results of a two-year programme for the early optimisation of antibiotic treatment in patients admitted to the Costa del Sol Hospital (Marbella. Spain).

**Methods:**

A prospective two-year cohort study was conducted, evaluating all episodes of bacteraemia at the Costa del Sol Hospital. Epidemiological and microbiological characteristics, any modification of the initial antibiotic treatment, prognostic risk stratification, early mortality related to the episode of bacteraemia, and mortality after the seventh day, were included in the analysis.

**Results:**

Seven hundred seventy-three episodes of bacteraemia were treated, 61.6% males and 38.4% females. The mean age was 65.2 years. The condition was most commonly acquired in the community (41.4%). The bacteraemia was most frequently urological in nature (30.5%), and *E coli* was the microorganism most frequently isolated (31.6%). In 51.1% of the episodes, a modification was made to optimise the treatment. In the first week, 8.2% died from bacteraemia, and 4.5% had died when they were located. The highest rates of death were associated with older patients, nosocomial acquisition, no source, McCabe score rapidly fatal, Charlson index ≥3, Pitt index ≥3 and treatment remained unmodified.

**Conclusion:**

The existence of bacteraemia control programmes and teams composed of clinicians who are experienced in the treatment of infectious diseases, can improve the disease outcome by enabling more severe episodes of bacteraemia to be recognised and their empirical treatment optimised.

## Background

Bacteraemia is a major cause of morbidity and mortality in patients admitted to hospital [1–3]. Many of the deaths caused by this infectious process take place in the first few hours following the onset of bacteraemia. For this reason, it is crucial to implement measures for diagnosis and treatment as soon as possible [4].It is also particularly important to identify the subgroup of patients who are most likely to present a poor prognosis and therefore suffer greater mortality. Recent studies have reported a higher incidence of mortality among patients who receive inappropriate empirical therapy in the initial hours of bacteraemia [5].

The aim of this study is to describe the results achieved by a multidisciplinary early intervention programme for patients with bacteraemia, and its impact on attributable mortality during the first 7 days. In additions, we aimed to determine risk factors for 7 day mortality.

## Methods

The Costa del Sol Hospital (HCS) is a general hospital of specialities that forms part of the public health system in Andalusia (southern Spain). It has 350 beds and functions as a referral hospital for 396,012 inhabitants of the Western Costa del Sol (Málaga province). The software used is HP Doctor, an operating system that enables administrators to work with a single computerised medical record.

Since 2008, the HCS has employed a multidisciplinary programme for early intervention in patients with positive blood cultures (except the ICU and the paediatrics department), staffed by clinicians from the infectious disease and microbiology groups [6, 7]. Positive blood cultures are notified at an early stage by the microbiology service group, at a joint meeting with experts in the treatment of infectious diseases. Subsequently, a systematic search is conducted to identify hospitalised patients with bacteraemia and the doctor treating them. The same process is used to locate patients who have been sent home from the emergency department, via an action protocol based on clinical status and treatment recommended at the moment of discharge [8]. With these data available, an interview is held with the physician responsible (in the case of hospitalised patients), to assess the outlook for the optimisation of antibiotic treatment, taking into account the antimicrobial spectrum, the drug dose and Pk/Pd parameters.

The first action taken by the bacteraemia control team is based on the results of the Gram stain of blood cultures, facilitated by the team’s microbiologist. The second stage of the procedure is applied after the definitive identification of the microorganism producing the bacteraemia and of its sensitivity profile. At this point, the empirical treatment is confirmed or assessed according to the microbiological results. In addition, a recommendation is made regarding the duration of antibiotic treatment. The outcome of the intervention, after an interview with the doctor responsible for follow up, is transcribed onto a non-binding clinical report included in the patient’s computerised medical history.

A prospective cohort study was conducted to evaluate the results of an early intervention programme developed by the HCS Infectious Diseases and Microbiology group for patients aged 14 years and older with bacteraemia. The following variables were analysed: age, sex, microbiological isolates, source of infection acquisition, source of origin and evolution of condition in response to the modified antimicrobial treatment. The condition was considered to be of community origin in patients with no previous hospitalisation or contact with health care services; or nosocomial, in accordance with CDC criteria or treatment-related, under the definition proposed by Friedman [9]. The prognosis of the underlying disease was defined using the criteria of McCabe and Jackson, with the following classifications: rapidly fatal (RF), ultimately fatal (UF) or nonfatal (NF). Mortality was predicted using Charlson’s index of comorbidity [10] and Pitt’s index of bacteraemia [11].

True bacteraemia was defined as the isolation of one or more clinically-apparent bacteria or fungi in the blood cultures [12]. The presence of coagulase-negative *Staphylococcus* (ECN), *Streptococcus viridans*, *Corynebacterium* spp., *Propionibacterium* spp. or *Bacillus* spp. in just one blood culture bottle was considered to result from contamination. Polymicrobial bacteraemias were not excluded. The patients were not included in more than one occasion.

### Statistical analysis

A descriptive analysis was performed, with measures of central tendency and dispersion for the quantitative variables and of frequency distribution for the qualitative ones, together with 95% confidence intervals for the outcome variables. Taking as the variable for the comparison of subgroups the presence or absence of mortality at the conclusion of the study, a bivariate analysis was performed using the chi-square test for qualitative independent variables (or Fisher’s exact test if fewer than five observations were expected), and Student’s t test for quantitative independent variables (or the Mann-Whitney U test in the case of non-normal distribution, which was checked by the Kolmogorov-Smirnov test). Finally, three multivariate logistic regression models were constructed, taking as the outcome variable the mortality at the conclusion of the study, and including the independent variables by forward stepwise selection, including the relative risk with the corresponding 95% CI. In each multivariate model, each of the comorbidity indicators to be evaluated was introduced independently. In all analyses, the limit of statistical significance was established at *p* < 0.05.

## Results

### “Analysis of the entire cohort”

Over a period of 2 years, 773 episodes of bacteraemia aged 14 years or more were evaluated. Characteristics of patients and infection are presented in Table 1. The gender distribution was 476 males (61.6%) and 297 females (38.4%). The mean age of the patients was 65.2 years (SD 16.7). Infection acquisition took place in the community in 320 episodes (41.4%), was healthcare-associated in 280 (36.2%) and was nosocomial in 173 (22.4%). The severity of comorbidities was determined by the McCabe index (NF: 43.7% UF: 41.2%, RF: 15.2%) and the Charlson index (≤2: 54.8%; >2: 45.2%). The severity of bacteraemia was determined by the Pitt index (≤2: 83.3%; >2: 16.7%). Although 45% of the patients had a significantly high Charlson comorbidity index >2, only a relatively small proportion (16.7%) of the patients with bacteraemia had an increased prognosis of mortality in 30 days (Pitt index ≥3).

**Table 1:** Descriptive analysis of the study sample (773 patients)

Variables	Total	
	*n*	%
Sex		
Male	476	61.6
Female	297	38.4
Age (years)		
Mean – SD	65.2	16.7
Isolate		
*E. coli*	246	31.8
ECN	172	22.3
*Klebsiella sp*	59	7.6
MSSA	52	6.7
*Pseudomonas aeruginosa*	27	3.5
*Enterobacter. sp*	22	2.8
*Proteus.sp*	20	2.6
*Enterococcus faecalis*	19	2.5
*Candida sp*	10	1.3
MRSA	13	1.7
Other	115	14.9
*Streptococcus pneumoniae*	18	2.3
ESBL multi-resistance		
Absent	736	95.2
Present	37	4.8
Type of infection		
Community-acquired	320	41.4
Nosocomial	173	22.4
healthcare-associated	280	36.2
Source of infection		
Absent	63	8.2
Present	710	91.8
Treatment modification		
Change to another drug	395	51.1
Extension of duration	28	3.6
No change	350	45.3
McCabe^a^		
NF	337	43.7
UF	318	41.2
RF	117	15.2
Pitt^a^		
0–2	643	83.3
≥ 3	129	16.7
Charlson^a^		
0–2	423	54.8
≥ 3	349	45.2

^a^One patient lost to analysis

The main source of origin of the bacteraemia were urological (*n* = 217; 30.5%), abdominal (*n* = 160; 20.7%). The rest of the sources are in Table 1. The most frequently isolated microorganisms were *E. coli* (246 episodes; 31.6%), *Klebsiella* sp. (59 episodes; 7.6%), *Enterobacter* sp. (22 episodes; 2.8%), *Proteus mirabilis* (20 episodes; 2.6%), *Pseudomonas aeruginosa* (27 episodes; 3.5%), *Staphylococcus* coagulase-negative (172 episodes; 22.3%), *Staphylococcus aureus* (65 episodes; 52 (6.7%) of which were MSSA and 13 (1.7%) were MRSA), *Enterococcus faecalis* (19 episodes; 2.5%), *Enterococcus faecium* (9 episodes; 1.2%), anaerobes (17 episodes; 2.3%), and *Candida* sp. (10 episodes; 1.3%). Enterobacteriaceae producing extended spectrum beta-lactamase originated 37 episodes (4.8%). The cases provoked by coagulase-negative *Staphylococcus* were related to health care in patients with central or tunnelled catheters or haemodialysis. Only 4 episodes of bacteraemia were polymicrobial and they have been included in the section of others (table n° 1).

Of the 773 episodes of bacteraemia, 21 were excluded from the analysis of mortality because the patient was transferred to another centre, and so 752 were finally evaluated. Of these 111 died (14.7%; 95% CI: 12.2–17.4). Thirty-four patients (4.5%) had died before they were located. In the first week of hospitalization, 62 patients (8.2%) died with the cause of death attributed to bacteraemia. During the first 30 days, another 49 patients (6.5%) died for reasons not directly attributable to bacteraemia after the first week. Six hundred forty-one patients (85.2%) survived more than 30 days.

Regarding modifications to the antimicrobial treatment applied in these episodes of bacteraemia, in 350 cases (45.3%) there was no such modification, in 27 cases (3.6%) the treatment duration was extended and in 396 (51.1%) a modification was made to optimise the treatment. In the 51,1% of cases, the antimicrobial spectrum was enlarged due to the inadequate empirical treatment by switching to other drugs (see Table 1).

The bivariate analysis revealed significant differences (*p* < 0.001) between mortality attributable to bacteraemia and age (the differences were greater for older patients), the McCabe index with ultimately fatal (UF) and rapidly fatal (RF) disease, the Charlson and Pitt index score ≥ 3, the absence of a source of origin and the non-modification of treatment. There were no statistically significant differences regarding gender distribution, microorganism isolated or place of infection acquisition, although nosocomial acquisition presented a higher percentage (18%; 30 patients) than community-acquired infection (13.2%; 41 patients) and healthcare-associated infection (14.6%; 40 patients) (see Table 2).

**Table 2:** Bivariate analysis with respect to first-week mortality

Variables	Alive		Died		*p*
	*n*	%	*n*	%	
Sex					
Male	389	84.0	74	16.0	0.276
Female	252	87.2	37	12.8	
Age (years)					
Mean – SD	64.1	17.1	71.2	13.1	<0.001
ESBL multi-resistance					
Absent	609	85.1	107	14.9	0.695
Present	32	88.9	4	11.1	
Type of infection					
Community-acquired	270	86.8	41	13.2	0.371
Nosocomial	137	82.0	30	18.0	
healthcare-associated	234	85.4	40	14.6	
Source of infection					
Absent	45	73.8	16	26.2	0.014
Present	596	86.3	95	13.7	
Treatment modification					
Change to another drug	343	89.3	41	10.7	<0.001
Extension of duration	27	96.4	1	3.6	
No change	271	79.7	69	20.3	
McCabe					
NF	314	95.4	15	4.6	<0.001
UF	241	78.8	65	21.2	
RF	86	74.1	30	25.9	
Pitt					
0–2	569	90.6	59	9.4	<0.001
≥ 3	72	58.5	51	41.5	
Charlson					
0–2	374	91.4	35	8.6	<0.001
≥ 3	267	78,1	75	21.9	

Each of the multivariate logistic regression models, differentiated by the comorbidity adjustment applied, includes the increased risk of mortality as an associated variable. The model incorporating McCabe category RF is associated with a greater adjusted risk of death at 7 days (RR 7.616, 95%CI: 3.81–15.221) (Table 3). In the Pitt-adjusted multivariate model (Table 4) for category ≥3, the RR is 7.014 (95%CI: 4.345–11.321), compared with RR = 2.678 (95%CI: 1.706–4.204) for category ≥3 on the model with Charlson adjustment (Table 5). In the three multivariate models, age, absence of a source of infection and the non-modification of treatment are all risk factors for greater mortality, while the model in which comorbidity is adjusted by the Charlson index also includes the presence of nosocomial infection.

**Table 3:** Multivariate logistic regression of first-week mortality with comorbidity adjustment by McCabe

Variables	β	*p*	Relative risk	95% C.I.	
				Lower	Upper
Age	0.026	0.001	1.026	1.010	1.042
Source of infection					
Absent			1.000		
Present	1.061	0.003	2.889	1.440	5.796
Treatment modification					
Change to another drug		0.001	1.000		
Extension of duration	−0.426	0.691	0.653	0.080	5.328
No change	0.840	<0.001	2.316	1.482	3.618
McCabe					
NF		<0.001	1.000		
UF	1.668	<0.001	5.304	2.896	9.714
RF	2.030	<0.001	7.616	3.810	15.221

**Table 4:** Multivariate logistic regression of first-week mortality with comorbidity adjustment by Pitt

Variables	β	*p*	Relative risk	95% C.I.	
				Lower	Superior
Age	0.028	<0.001	1.028	1.013	1.044
Source of infection					
Present			1.000		
Absent	1.016	0.004	2.761	1.379	5.528
Treatment modification					
Change to another drug		<0.001	1.000		
Extension of duration	−0.566	0.591	0.568	0.072	4.476
No change	0.893	<0.001	2.442	1.539	3.874
Pitt					
0–2			1.000		
≥ 3	1.948	<0.001	7.014	4.345	11.321

**Table 5:** Multivariate logistic regression of first-week mortality with comorbidity adjustment by Charlson

Variables	β	*p*	Relative risk	95% C.I.	
				Lower	Lower
Age	0.028	<0.001	1.029	1.012	1.045
Type of infection					
Treatment-related		0.038	1.000		
healthcare-associated	−0.054	0.833	0.947	0.572	1.568
Nosocomial	0.625	0.029	1.868	1.065	3.276
Source of infection					
Absent			1.000		
Present	0.892	0.009	2.440	1.252	4.756
Treatment modification					
Change to another drug		<0.001	1.000		
Extension of duration	−0.938	0.382	0.391	0.048	3.210
No change	0.911	<0.001	2.487	1.585	3.904
Charlson					
0–2			1.000		
≥ 3	0.985	<0.001	2.678	1.706	4.204

### “Analysis of patients discharged from the emergency department”

Ninety patients were located at home. Of these 90, the median age was 67 years, and they comprised 54 males (60%) and 36 females. Infection acquisition took place in the community in 51 cases (56.6%) and the microorganism that was most frequently involved was *Escherichia coli*, with 31 cases (34.4%). The median score on both the Charlson index and the Pitt index of bacteraemia was 1. Thirty-eight cases (42.2%) required hospitalisation. A Pitt index >1 and treatment modification were the variables most frequently associated with the need for hospital admission. One patient (1.2%) died and 6 (6.6%) could not be located.

## Discussion

Bacteraemia remains a significant cause of morbidity and mortality in hospitalised patients, despite recent advances in diagnosis and treatment strategies [13, 14]. The recognition of episodes of bacteraemia according to criteria of severity and early treatment optimisation can facilitate actions to improve final outcomes. The administration of inappropriate antibiotic therapy is an independent predictor of mortality in patients with bacteraemia and sepsis [5]. The existence of multidisciplinary teams, composed of experts in the field of infectious diseases, together with pharmacists and microbiologists, enables a comprehensive approach to be taken to antibiotic treatment, producing not only better and earlier diagnosis but also enhancing outcomes in terms of Pk/Pd. The incorporation of pharmaceutical experts can also lend added value to the recommendations made by the bacteraemia control team [15]. Several reports have been issued on the reductions achieved in the mortality of certain infectious diseases when the treatment strategy is directed by experts in this field [16].

This paper describes the results obtained from the application, over 2 years, of a multidisciplinary programme for patients with bacteraemia, at a general hospital of specialities in Andalusia (Spain). Of the 773 episodes considered, 40% were community-acquired; the most frequent source of infection was urological and the microorganism producing the bacteraemia was *E coli* in 30.6% of cases, which is in accordance with previous reports [17].

Fewer than 5% of patients died within 24 h, before the bacteraemia control team was able to intervene. A further 8.2% of the patients died within the first 7 days of hospital admission, due to the bacteraemia episode. In 51.1% of the episodes, the treatment was modified in order to optimise its antimicrobial impact.

Older patients, the nosocomial acquisition of bacteraemia and the absence of identifiable source of infection are all associated with increased patient mortality. These findings are consistent with previous research in the field. Thus, a multicentre study, conducted in Andalusia (Spain), reported that mortality at days 14 and 28, in elderly patients, was associated with severe forms of infection and with inadequate empirical treatment [18]. Another study reported higher mortality in bacteraemia cases of nosocomial origin, possibly interacting with resistant organisms and in conjunction with inadequate treatment [19]. The greater mortality of patients with no identifiable source of infection is probably accounted for by the increased difficulty this causes in establishing appropriate empirical treatment and by the impossibility of acting on or removing an initial source of bacteraemia. Recently, Mansur et al. [20] reported increased mortality among critically-ill patients with primary bacteraemia versus those with bacteraemia of respiratory or abdominal origin. Patients with bacteraemia of unknown origin presented a higher SOFA score than those with a respiratory or abdominal source of infection.

Patients with high scores on the risk stratification indices for mortality prediction, such as McCabe RF (RR: 7.616), Pitt’s bacteraemia index ≥3 (RR: 7.019) and Charlson’s comorbidity index ≥3 (RR: 2.68), were more liable to suffer early mortality (in the first 7 days), as reported elsewhere [21]. Both the presence of comorbidity or chronic debilitating disease, as measured by the Charlson index, and the presentation of severe forms of infection, often with haemodynamic instability, may account for the higher mortality recorded [22].

The main limitation of the present study is that, although it was prospective, we did not know the incidence of mortality before the bacteraemia control team began its work, since our hospital classifies cases by disease process, and the study cases were coded as sepsis, not bacteraemia. For this reason, our results cannot be compared with previous data. Nevertheless, they can be compared with those from general hospitals of specialities that attend patients with the same profile of complexity. The continuing application of the early care programme for patients with bacteraemia will enable a long-term assessment to be made of outcomes, and may reduce morbidity and mortality among these patients.

We believe that early intervention in patients with a strong probability of presenting bacteraemia, based on multidisciplinary programmes integrated into routine clinical practice, would enable clinicians to stratify the severity of the process and facilitate rapid optimisation of empirical antimicrobial therapy in the initial response and focused treatment thereafter. This set of actions could reduce morbidity and mortality in patients with bacteraemia. Additionally, early intervention programmes for patients with bacteraemia would enable the recovery of patients who have been discharged from the emergency department and who require treatment modification or even hospitalisation [8].

## Conclusions

Our program of identification and early action on patients with bacteraemia allowed the localization of both hospitalized patients and those who discharged from the Emergency Department, who had a higher risk of unfavorable outcome based on the McCabe and Charlson comorbidities indexes and Pitt bacteraemia index. The bacteraemia control team optimized antimicrobial treatment in slightly more than half of the cases, being this a favourable influence in the final mortality of the patients.

## Abbreviations

95% CI: 95% confidence interval
CDC: Center for diseases control
HCS: Costa del Sol Hospital
ICU: Intensive care unit
NF: Nonfatal
RF: Rapidly fatal
RR: Relative risk
SD: Standard deviation
SOFA score: Sequential organ failure assessment score
UF: Ultimately fatal
